# Evaluating the Sealing Performance of Endodontic Sealers: Insights Into Achieving Complete Sealing

**DOI:** 10.7759/cureus.71967

**Published:** 2024-10-20

**Authors:** Ajay Chhabra, Ramya K P., Saravana Prathap, Priyanka Yadav, Himani Mehra, Sona J Parvathy

**Affiliations:** 1 Conservative Dentistry and Endodontics, All India Institute of Medical Sciences, Kalyani, Kalyani, IND; 2 Dentistry, All India Institute of Medical Sciences, Kalyani, Kalyani, IND

**Keywords:** calcium silicate, epoxy resin, marginal adaptation, mineral infiltration zone, silicone

## Abstract

Background

The importance of achieving optimal sealing in endodontic procedures cannot be overstated, as it directly influences the success and durability of root canal treatments. The objective of this research was to measure and identify the sealing ability of endodontic sealers by evaluating their adhesion to root canal dentin and the extent to which they achieve a complete seal.

Methods

A total of 60 extracted lower premolar teeth were randomly divided into three groups of 20 samples each. After root canal preparation with Neo Endo Flex files, each group was treated with a different sealer: calcium silicate-based (Bio-C Sealer), epoxy resin-based (Seal Pex), and silicone-based (Roeko Gutta Flow 2). The sealers were applied using the single cone obturation technique with gutta-percha points. The samples were stored at 37°C and 100% humidity for one week. Finally, the teeth were sectioned longitudinally and analyzed using scanning electron microscopy (SEM) to assess marginal adaptation.

Results

The largest gaps were found in group III (Roeko Gutta Flow 2) (86.2 µm coronal, 91.1 µm apical) and the smallest in group I (Bio-C Sealer) (52.5 µm coronal, 53.7 µm apical). Significant differences were confirmed using one-way ANOVA and Tukey's post hoc tests, with group I having the best adaptation and group III the poorest. Under SEM, the coronal halves in every group outperformed the apical halves in terms of adaptability.

Conclusion

The study found that coronal halves adapted better than apical halves, and Bio-C Sealer performed better than Seal Pex and Roeko Gutta Flow 2. These findings indicate that Bio-C Sealer may improve sealing and decrease microleakage risk in endodontic procedures. This study needs further research and evaluation of the long-term effects of Bio-C Sealer in clinical practice.

## Introduction

Endodontic treatment is a series of essential steps to restore the health and function of a tooth affected by pulp disease or injury. This includes carefully removing infected or necrotic pulpal tissue, root canal contouring and filing to provide proper tooth canal shape and cleaning, as well as obturating the root canals with appropriate materials, and, finally, placing a permanent restoration to protect the tooth and restore its fundamental function. The high success rate for endodontic therapy has been demonstrated in research, usually between 85% and 95% [1]. However, inadequate obturation is a major contributor to endodontic failures, with approximately 58% of these failures being attributable to this factor [2]. Multiple factors can lead to insufficient obturation, including poor obturation techniques and inadequate instrumentation during the cleaning and shaping phases of treatment [3].

Due to the adhesion and dimensional changes of the core obturating material gutta-percha, complete obturation is often not achieved. Although gutta-percha has favorable properties, it is commonly used in endodontics; however, it is often not sufficient by itself to produce an adequate seal. The use of gutta-percha in conjunction with a sealer cement that can securely bond to both root dentin and gutta-percha itself is necessary to improve the sealing ability of the filling [4]. This combination enhances obturability, decreases the risk of microleakage, and improves the long-term success of endodontic therapy. Properly selected and used sealers can reduce the challenges of incomplete obturation, leading to more successful outcomes.

The passage of sealers into dentinal tubules may be impacted by the smear layer cutting, which could have clinical implications [5]. The earlier protocols were frequently characterized by a focus on the radiographic apex of the filling, which tended to divert attention from the more important aspect of three-dimensional root canal obturation. This comprehensive sealing is a key factor for the long-term success of root canal treatment. Microleakage at the root canal, resulting from poor sealing of the root canal walls and the core obturating material, is an important factor in the failure of endodontic treatment [6]. Therefore, to achieve efficient sealing, one must pay attention to those details at the interfaces, emphasizing the need for precision and thoroughness in the obturation. Thus, the modification of a sealer to the dentin is the key element determining root canal microleakage and reinfection [7].

The purpose of this study is to determine and compare the sealing ability of different endodontic sealers in achieving complete sealing of root canal systems. The study seeks to find out whether different sealers are effective in preventing microleakage and improving long-term treatment outcome by carrying out controlled in vitro experiments.

## Materials and methods

Collection of samples

For the present research, ethical clearance for the study was granted by the Institutional Ethics Committee of All India Institute of Medical Sciences Kalyani, Kalyani, West Bengal, India, in April 2024, in which 60 lower premolars were used. To remove any organic waste, the teeth were immersed in 2.5% NaOCl (sodium hypochlorite) for 48 hours. Afterward, they were rinsed under running water and stored in a saline solution until required. The extracted teeth were excluded from the study if they exhibited craze lines, severe curvatures, resorption, endodontic fillings, or insufficient apex development. The extracted teeth had visible debris and calculus, which were removed using ultrasound.

Processing of samples

An endo-access bur was used to prepare the access. Next, until the instrument's tip was barely visible at the apical foramen, a no. 10 K-file was advanced towards the apical part. The length of the file was measured, and then 1 mm was subtracted from that length to calculate the working length of the root canal. Neo Endo Flex files were used successively up to 25/04 to shape and clean the root canal, creating a root canal preparation that tapers gradually.

A 27-gauge side vented needle was used to irrigate each canal with 2 mL of 3.5% NaOCl after all files had been used, and then root canal treatment prep (17% EDTA [ethylenediaminetetraacetic acid] with carbamide peroxide) was applied for lubrication and chelation of the root canal. As a final rinse, normal saline was used to get rid of any left-out chemical irritants [8].

Depending on the type of sealer used, the samples were randomly split into three groups after the canals were dried using paper points (20 samples in each group). The following sealers were employed:

Group 1: calcium silicate-based sealer (Bio-C Sealer, Angelus, Londrina, Brazil)

Group 2: epoxy resin-based sealer (Seal Pex, Waldent, Delhi, India)

Group 3: silicone-based sealer (Roeko Gutta Flow 2, Coltene, Langenau, Germany)

All the teeth were obturated with gutta-percha points and an endodontic sealer using single-cone obturation techniques. The sealants were mixed according to the manufacturer's specifications. The gutta-percha cone was immersed in the sealer before it was slowly inserted into the canal. The instruments were repeatedly readjusted until maximum working length was achieved. The cone was later trimmed down to the level of the orifices. The obturation was considered optimal if the radiograph showed no voids. If, however, any voids were detected, re-obturation was carried out. Then, DenTemp was applied to the access cavities to seal them, for which it had been designed. One week later, the teeth were stored at 37°C and 100% humidity.

Assessing the marginal adaptation using scanning electron microscopy

Roots were marked into apical (0-5 mm) and coronal (8-13 mm) portions, sectioned longitudinally in the labiolingual direction. Sections underwent vacuum drying and platinum coating, and SEM analysis was performed by different examiners to avoid examiner bias.

Furthermore, the marginal adaptation of the sealers was evaluated using SEM at different magnifications such as 909x, 1.70Kx, and 1.32Kx. The images were analyzed to measure the gaps between the sealer and the canal wall. Statistical analysis was performed to compare the marginal adaptation of the three sealers using one-way ANOVA, with significance set at p < 0.05.

## Results

The results indicate that marginal adaptation was consistently better in the coronal halves compared to the apical halves across all groups. Group 1 demonstrated the best overall adaptation, with the smallest marginal gaps in both the coronal and apical thirds, showing more uniformity throughout the tooth. Group 2 exhibited slightly larger gaps than group 1, with a more noticeable difference between the coronal and apical regions. Group 3 had the largest marginal gaps, indicating the poorest adaptation, particularly in the apical third. Overall, group 1 achieved the best marginal sealing, while group 3 showed the most significant discrepancies (Table 1).

**Table 1:** Mean marginal gap measurement of various sealers at different levels

	Group 1 (µm)	Group 2 (µm)	Group 3 (µm)
Coronal third	52.5	56.1	86.2
Apical third	53.70	61	91.1

**Figure 1:**
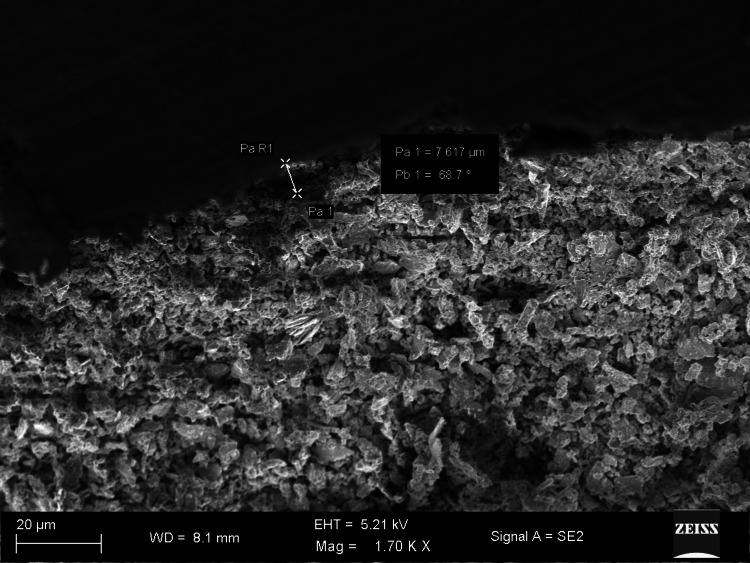
Picture of the coronal halves captured using scanning electron microscopy (Bio-C Sealer)

**Figure 2:**
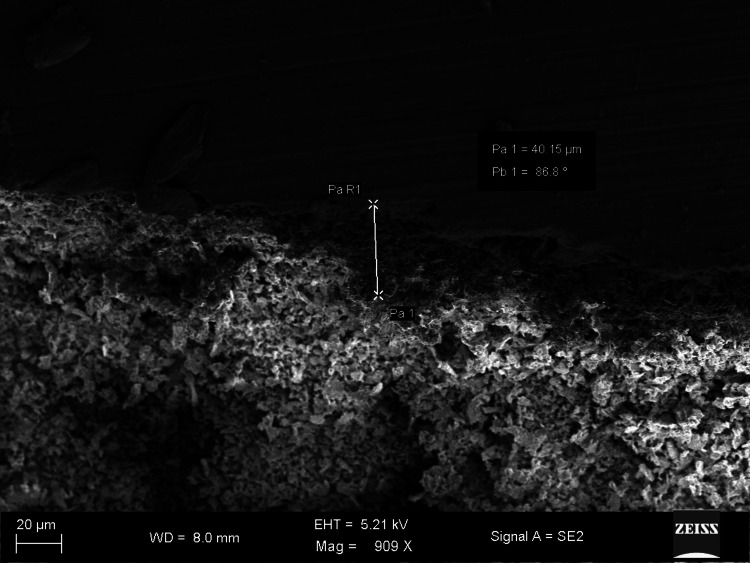
Picture of the coronal halves captured using scanning electron microscopy (Roeko Gutta Flow 2)

**Figure 3:**
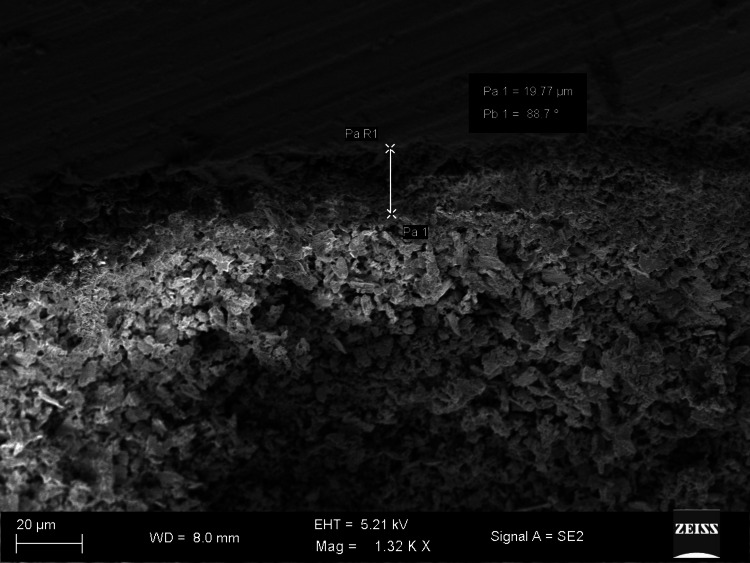
Picture of the coronal halves captured using scanning electron microscopy (Seal Pex)

The SEM images show differences in marginal gaps among the samples from each experimental group. Figure 1 reveals a marginal gap of 7.617 µm and an angle of 68.7°, showing very good adaptation (group 1). The largest measure of the marginal gap shown in Figure 2 is 40.15 µm and an angle of 86.8°, pointing to inadequate marginal adaptation (group 3). Group 2 illustrated in Figure 3 shows a wider opening of 19.77 µm and an angle of 88.7°, pointing to weaker adaptation than shown in the first image (group 1).

Statistical evaluation

The significant difference between the coronal third and apical third was evaluated using a one-way ANOVA measure across different categories of sealers. There was a normal distribution of outcome measurement across different categories as assessed by the Q-Q plot, and no outlier was assessed by the box-whisker plot. As determined by Levene statistics, considering that all variances are equal. The coronal third (75(2,57), p<0.001) and apical third (61.7(2,57), p<.001) measurements were significantly different across different categories (Table 2). A post hoc Tukey analysis showed the mean difference in the coronal third of groups 1 and 2 to be -3.63, and there was a statistically significant mean difference between groups 2 and 3 and groups 1 and 3 of -30.1 and -33.7, respectively. The mean difference in apical third between group 1 and group 2 was -3.63, whereas that between groups 2 and 3 and groups 1 and 3 were -33.7 and -30.1, respectively, which was statistically significant.

**Table 2:** Pairwise comparison of mean apical and coronal third measurement across different categories of sealers

	Group 1 (µm), mean ± SD	Group 2 (µm), mean ± SD	Group 3 (µm), mean ± SD	F (df)	p-Value	Group 1 vs group 2 p-value	Group 2 vs group 3 p-value	Group 1 c vs group 3 p-value
Coronal third	52.5 ± 8	56.1 ± 6.46	86.2 ± 5.53	75 (2,57)	<0.001	0.461	<0.001	<0.001
Apical third	53.7 ± 9.67	61 ± 7.27	91.1 ± 6.67	61.7 (2,57)	<0.001	0.121	<0.001	<0.001

## Discussion

The root canals are meticulously prepared to ensure the long-term therapeutic success. Three-dimensional root filling is recommended to avoid complications. The notion of an ideal apical seal prompted researchers to search for filling and sealing materials that are stable and biocompatible and offer a hermetic closure at the apical foramen. Improved penetration and adhesive properties offer two key benefits: first, they enhance sealing by increasing the surface contact between the sealer and the dentin, and, second, they contribute to an antimicrobial effect by entombing any remaining microorganisms within the dentinal tubules [9,10].

To assess the marginal adaptation to root dentin, the current research used three root canal sealers, namely Bio-C Sealer, Seal Pex, and Roeko Gutta Flow 2 sealer. Bio-C Sealer showed better marginal adaptation than all other sealers, and Roeko Gutta Flow 2 demonstrated subpar adaptation. All sealer types showed greater interfacial gaps at the apical portion than at the coronal portion of the canal (p<0.001). Fewer tubules, or tubules with smaller diameters or more frequently closed ones, are likely found in the apical root canal [11,12]. Additionally, the roots' apical region exhibits a noticeable structural diversity. For instance, the orientation and the density of primary dentinal tubules vary, and some regions are completely tubule-free [13-15]. Also, the apical part may have tissue that resembles cementum, which can occlude the tubules. The smear layer from that portion is also challenging and may serve as a physical barrier that hinders the sealer's ability to adhere to the root canal dentin [16]. Our results were consistent with research by Pawar et al. [17], who assessed the sealing capacity of different sealers. It was found that AH Plus sealer does not exhibit the same level of penetration to dentinal tubules as bioceramic sealer. Within the biological milieu, bioceramics are biocompatible, non-hazardous, and chemically stable [18] The manufacturer describes Bio-C Sealer, a calcium silicate sealer, as radiopaque, hydrophilic, resin-free, and biocompatible, with an average particle size of less than 2 µm. Due to its high pH, this sealer also has significant antibacterial properties [19].

Additionally, because of its higher flow rates, the sealer can penetrate the complexities in root canal networks [20]. The reason why Bio-C Sealer causes less leakage could be because of the mineral infiltration zone, an interfacial layer that forms between dentin and calcium-silicate-based cements, which is formed when the Bio-C Sealer comes into contact with moisture and tissue fluids. This interaction causes active ions to be released, which interact with the dentin's organic and inorganic matrix. A great biological seal is provided by this region of mineral infiltration in the dentin. The impact of the mineral infiltration zone on the endodontic treatment is unclear, and the outcome can be either positive or negative. Some researchers show the possible link, but scientific data are still limited, and thus more studies are needed to determine the actual influence of the factor on the treatment results and the prognosis [19]. The formation of calcite crystals is due to the interaction of calcium ions with the tissue's carbon dioxide, and this may positively affect the outcomes. These crystals can improve cement retention by decreasing porosity and marginal gaps [21,22]. On the other hand, due to its porous structure, apatite deposition in certain trials did not lessen leakage [23]. Studies have previously assessed the sealing effectiveness using a variety of microleakage techniques, including radioisotope tracing, fluid filtering, electrical approaches, dye penetration, and marginal adaptation by SEM [24]. SEM was used in this investigation to examine the marginal gaps. The benefit of employing SEM over other microleakage techniques is that it allows for the observation of flaws at the submicron level at the necessary magnification and also allows for the preservation of microphotographs for final evaluation. SEM provides glaringly clear images because it uses electromagnets instead of lenses, which provides the observer better control over the level of magnification [25,26].

Conversely, the Gutta Flow 2 sealer showed an increased gap. This may be attributed to the polydimethylsiloxane-based composition of Gutta Flow 2 sealer, which contains silicone. The silicone may lead to increased surface tension and poor wettability of the dentin, hindering the sealer’s ability to spread evenly. Additionally, the absence of a chemical bond between the sealer and the gutta-percha particles can result in a greater number of voids [27]. The authors hypothesized that an inferior seal could result from this thixotropic sealer flowing under the pressure of inserted gutta-percha particles between the root canal and cone during obturation. Given that endodontic sealers have been demonstrated to affect treatment outcomes, it is essential to investigate their properties and performance in clinical practice. The sealers used in this study are widely used, particularly Seal Pex and Bio-C sealers. This study is innovative since it compares and understands the current concepts of the use of different sealers. This information will largely determine the success of endodontic therapy.

Limitations of the study

This study demonstrated the sealing ability of different root canal sealers, but there are some limitations to be acknowledged. Artifacts, such as shredding or smearing of materials due to the sectioning of filled canals, may have affected marginal gap measurements. While SEM provides high-resolution imaging, it lacks dynamic views and may not adequately represent the dynamic sealing process or long-term performance of the sealers. The results may not be fully generalizable because in vitro conditions do not accurately replicate the clinical environment. Future research with larger sample sizes and in vivo testing is needed to validate these findings.

## Conclusions

The findings of this study are valuable in understanding the sealing abilities of three different endodontic sealers: Bio-C Sealer, Seal Pex, and Roeko Gutta Flow 2. The best marginal adaptation was found among these, with the smallest gaps in the coronal and apical thirds of the root canal for the calcium silicate-based Bio-C Sealer. On the other hand, gaps were largest in Roeko Gutta Flow 2, especially in the apical third, indicating its weaker sealing properties. Bio-C Sealer appears to perform better than the other materials tested, and it may be more successful in preventing microleakage and increasing the success of root canal treatments. Although the findings need to be interpreted with caution as a result of study design in vitro, which does not fully mimic real-life clinical conditions, the protocol is relevant, and large body of results obtained with a variety of strategies in the lab emphasizes the potential value of the target as a clinical intervention. Moreover, material shredding or smearing during sectioning may also have contributed to the observed results. Further research and clinical studies are warranted to confirm these results and explore the long-term effects of using Bio-C Sealer in endodontic practice.
